# Microbial Strains in Fermented Dairy: Unlocking Biofunctional Properties and Health Benefits

**DOI:** 10.1155/ijfo/6672700

**Published:** 2025-08-19

**Authors:** Anuradha Wijesekara, Viraj Weerasingha, Shishanthi Jayarathna, Janak K. Vidanarachchi, Hasitha Priyashantha

**Affiliations:** ^1^Department of Animal and Food Sciences, Faculty of Agriculture, Rajarata University of Sri Lanka, Anuradhapura, Sri Lanka; ^2^Department of Molecular Sciences, Swedish University of Agricultural Sciences, Uppsala, Sweden; ^3^Department of Animal Science, Faculty of Agriculture, University of Peradeniya, Peradeniya, Sri Lanka

**Keywords:** bioavailability and antioxidant effects, fermentation methods and safety, gut and metabolic health, lactic acid bacteria, probiotics

## Abstract

Fermented dairy products like yoghurt, cheese, and kefir are essential for human nutrition and health. They offer a wide range of biofunctional properties, providing unique flavors along with substantial nutritional and therapeutic benefits. The inclusion of functional microorganisms, including probiotics, in dairy products offers a nutrient-dense matrix that promotes microbial viability, ensuring health advantages. This review explores the impact of microbial strains on the biofunctional properties of fermented dairy products, focusing on their contributions to gut and cardiovascular health, cancer risk reduction, bone density enhancement, weight management, and diabetes control. Special emphasis is placed on fermentation processes involving lactic acid bacteria, particularly their roles in safety assurance and preservation of product quality. This review emphasizes the antimicrobial, hypocholesterolemic, and antioxidant effects of cultured dairy products, highlighting their ability to improve bioavailability and health outcomes. In conclusion, fermented dairy products possess exceptional biofunctional properties that promote human health. To maximize their therapeutic potential for various medical conditions, further research into fermentation methods, microbial strains, and the underlying mechanisms is essential.

## 1. Introduction

Milk is a complex and dynamic fluid produced by the mammary glands of mammals, which is also highly nutritious. From the beginning of food cultures, humans consumed milk produced by different mammals. Milk is fermented into lactic acid by specific lactic acid bacteria (LABs), utilizing the availability and accessibility of suitable and harmless microorganisms [1]. They are a diverse group of bacteria that are mostly anaerobic, Gram-positive, resistant to acid, and do not produce spores [2]. The objectives of fermentation are to enhance the nutritional bioavailability and to impart a unique taste and aroma [3, 4]. Consisting of 314 species, *Lactobacillus* is the biggest genus among LABs, while *Streptococcus* (185), *Enterococcus* (70), *Aerococcus* (30), *Oenococcus* (5), *Leuconostoc* (28), *Weissella* (25), *Carnobacterium* (14), *Sporolactobacillus* (12), *Tetragenococcus* (5), *Lactococcus* (23), *Vagococcus* (19), and *Pediococcus* (16) are the other significant LAB genera with given respective numbers of species [2]. Alongside traditional starter cultures, many probiotic bacteria such as *Lactobacillus acidophilus*, *Lactobacillus casei*, and various *Bifidobacteria*, which are part of the LAB group, are commonly used in milk fermentation [2].

In sighting fermentation, it is generally considered a safe and acceptable preservation technology of food. Lactic acid, which is produced during fermentation, is responsible for the development of the unique body and texture of fermented milk products. Other formed organic acids, such as diacetyl, acetaldehyde, and acetic acid, contribute to the flavor and aroma of the final product. Together, they undoubtedly produce good quality products (Table 1) with highly appreciated organoleptic attributes.

Depending on their temperature tolerance, LABs used in dairy fermentation are broadly categorized into mesophilic and thermophilic groups [18]. Mesophilic strains prefer temperatures between 20°C and 30°C, whereas thermophilic strains grow optimally at 30°C–45°C. Further, they can also be categorized based on the end-products they generate during glucose fermentation. Homofermentative LABs such as *Streptococcus*, *Pediococcus*, and *Lactococcus* primarily produce lactic acid as their only metabolic product. In contrast, heterofermentative LABs, including *Weissella* and *Leuconostoc*, generate equal amounts of lactate, carbon dioxide (CO_2_), and ethanol during fermentation [19–21].

**Figure 1:**
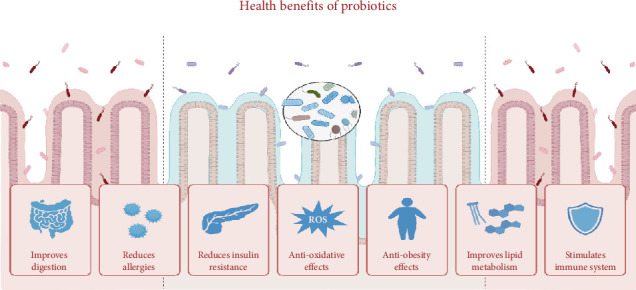
The health benefits of probiotics in fermented dairy products.

Use of LAB in dairy products offers biological functions and therapeutic benefits (Figure 1) that are closely linked to the metabolites produced throughout the fermentation process [22].

Decades of research suggest that the regular consumption of various fermented dairy products such as yoghurt, dairy curds, kefir, fermented cheeses, cultured buttermilk, kumis, and sour cream [23, 24] offers notable health benefits. The specific strains of LAB used, as well as the type of milk fermented, can influence outcomes related to gut and cardiovascular health, cancer risk reduction, bone strength, weight control, and diabetes management. Additionally, these products are recognized for their roles in lowering cholesterol levels and providing antioxidant effects [25]. While the biofunctional properties of fermented dairy products have been widely studied, the interplay between microbial strains and milk types remains underexplored. Investigating the unique characteristics of these products and their implications for human health is essential, particularly regarding their therapeutic potential. Although numerous studies have examined individual aspects of fermented milk products, there is a lack of comprehensive reviews synthesizing these findings. This review is aimed at addressing this gap by consolidating and analyzing the collective biofunctional properties and health benefits of fermented dairy products, providing a holistic perspective on their impact.

## 2. Biofunctional Properties of Fermented Dairy Products

### 2.1. Antioxidant Properties

**Figure 2:**
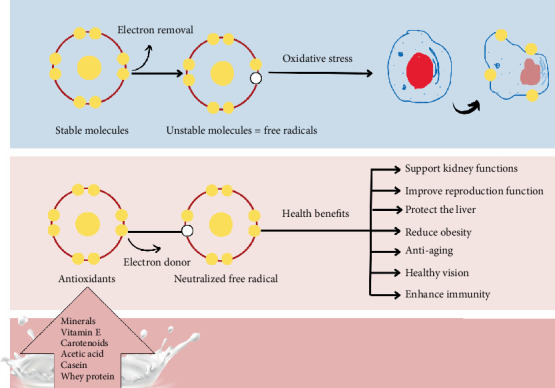
The mechanisms proposed for the antioxidant properties of milk and dairy products. *Source:* [26, 27].

Oxidative stress, triggered by the uncontrolled production of free radicals, poses a significant risk factor for various health conditions, including cardiovascular diseases, cancer, and neurodegenerative disorders. This subsection explores the antioxidant properties of milk and dairy products, which play a crucial role in neutralizing these reactive radicals, thus mitigating oxidative damage. Oxidation leads to the production of free radicals from the oxygen molecules. Those reactive radicals consist of unpaired electrons and stay as singles in the outermost orbit [26]. Uncontrolled generation of free radicals causes oxidative stress, which results in atherosclerosis, diabetes, accelerated aging, cardiovascular diseases [27], cancer, reproductive disorders, infertility, Parkinson's, and neurodegenerative diseases [26]. Antioxidants can neutralize and eliminate reactive radicals before they reach and harm cells [27]. According to Khan et al. [27], the antioxidant capacity of milk and dairy products is due to sulfur-containing amino acids cysteine, vitamins A and E, carotenoids, superoxide dismutase, catalase, and glutathione peroxidase (Figure 2).

Gjorgievski et al. [28] evaluated the antioxidant capacity of yoghurt made from sterilized whole cow's milk using different cultures, including mixed culture yoghurts with the symbiosis of *Streptococcus thermophilus* and *Lactobacillus delbrueckii* subsp. *bulgaricus* and monoculture yoghurts with *L. casei*, *L. acidophilus*, and *Bifidobacterium bifidus.* Among the tested strains, *L. acidophilus*-fermented milk has exhibited the highest antioxidant activity, achieving 54.86% free radical neutralization. In comparison, the lowest activity (45.17%) has been observed in milk fermented with the symbiotic culture. Supporting the antioxidant capabilities of LAB, Zhang et al. [29] stated that *L. casei* subsp. *casei* SY13 and *L. delbrueckii* subsp. *bulgaricus* LJJ also demonstrated strong antioxidant properties, effectively inhibiting linoleic acid peroxidation by 62.95% and 66.16%, respectively. Virtanen et al. [30] also showed the accordance of the above antioxidant results in milk whey during fermentation with LABs. As per them, the antioxidant activity of *L. acidophilus* is 42%, while *L. casei* resulted in 4%. Moreover, these authors studied the antioxidant properties of the symbiosis between *L. acidophilus* and *Bifidobacterium infantis*, stating an average of 27% antioxidant capacity. Further, Zhang et al. [29] stated a higher capacity for antioxidant value in plain yoghurts compared to kefir and buttermilk using the ferric reducing antioxidant power (FRAP) method. According to the authors, it is due to the fortification of milk powder and/or milk proteins used in yoghurt production. Many reports have declared that milk proteins, especially those rich in sulfur, hydrophobic amino acids, and whey proteins, are potent antioxidant substances [31]. Moreover, the literature [32] mentions the addition of chocolate, coffee, green tea extract, and dark-colored fruits (bilberries, forest fruits, blackcurrants, cherries, and strawberries) as flavorings that have imparted strong antioxidant properties to the product.

**Figure 3:**
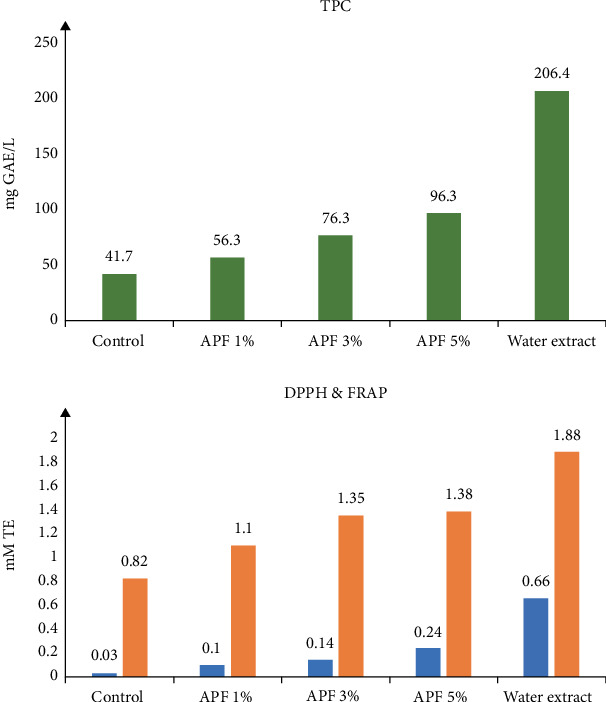
Antioxidant capacity of probiotic yoghurts with and without apple pomace flour and water extract measured using various methods. APF = apple pomace flour, 1 = yoghurt made with 1% of APF, 3 = yoghurt made with 3% APF, 5 = yoghurt made with 5% of APF, TPC = total polyphenolic content, DPPH = 2,2-diphenyl-1-picrylhydrazyl, FRAP = ferric reducing antioxidant potential. *Source:* [31].

Jovanović et al. [33] observed a strong correlation between total phenolic compounds (TPCs) and both DPPH and FRAP values in probiotic yoghurt added with apple pomace flour after inoculation with *L. acidophilus*, *S. thermophilus*, and *Bifidobacterium bifidum*. In addition, they concluded that apple peel extract had a high content of TPC and exhibited strong dose-dependent antioxidant activity (Figure 3).

Wijesekara et al. [34] reported that the stirred yoghurt inoculated with *S. thermophilus*, *Lactobacillus bulgaricus*, and *B. bifidus* and added natural plant colorants (10% *Hibiscus*, 4% turmeric, 6% spinach, and 4% blue pea over 14 days at 4°C) showed higher antioxidant properties compared to the control on Day 1 in parallel to the results obtained for TPC. This shows that natural phenolics boost antioxidant properties more than plain fermented dairy products.

According to a study by Chen et al. [24], cheddar cheese manufactured by adding adjunct probiotic microorganisms, *L. casei* and *Lactobacillus plantarum*, has improved antioxidant capabilities. They used several treatments: lactococci (1.5% *v*/*v*) as the control; lactococci (1.5% *v*/*v*) and *L. casei* (1.2% *v*/*v*); lactococci (1.5% *v*/*v*) and *L. plantarum* (1.2% *v*/*v*); and lactococci (1.5% *v*/*v*), *L. casei* (0.6% *v*/*v*), and *L. plantarum* (0.6% *v*/*v*). All of the above-mentioned cheddar cheese treatments reached their peak antioxidant activity in the 16^th^ week with values of 47.30%, 48.65%, and 51.72%, respectively, compared to the control sample. Supporting the above results, Table 2 represents the antioxidant capacities of cow cheddar cheese (CCC) and buffalo cheddar cheese (BCC) inoculated with *Streptococcus lactis* subsp. *lactis* and *S. lactis* subsp. *cremoris* under both standard and accelerated ripening conditions [35].

Considering the data given in Table 1, the total acidity content (TAC) of buffalo cheddar (73.91%), both fresh and aged, was found to be higher than that of cow cheddar (53.42%), after 120 days of traditional ripening, but both cheddar cheeses' antioxidant capacities increased over storage [35]. According to the authors, the increased levels of vitamin E, catalase, sulfur-containing amino acids, and glutathione peroxidase activities in buffalo cheddar may be responsible for the higher TAC. Gupta et al. [36] provide further evidence for the aforementioned study on the antioxidant capabilities of cheddar cheese. In the same study, the TAC value of cheddar cheese made from cow's milk increased to 70%, while the same rate for BCC reached 51.66% after 80 days.

Ozcan et al. [37] stated that kefir is manufactured by fermenting milk with kefir grains and/or by a commercial freeze-dried starter culture containing different species of LABs, acetic acid bacteria, yeasts, and mycelial fungi. They measured the antioxidant capacity of kefir samples made of buffalo milk treated with kefir grain (B-GR) and commercial starter cultures (B-SC) using ABTS and DPPH methods. During fermentation, the ABTS radical scavenging activity of B-GR was higher than that of B-SC at pH 4.6; yet in the end, B-SC had the highest ABTS activity at 11.86 mg Trolox/100 mL. Considering the results gained by the DPPH assay during the whole fermentation process, B-SC exhibited a higher DPPH radical scavenging activity than B-GR, except in the eighth hour of fermentation. A similar study has been done by Yilmaz-Ersan et al. [38] on the antioxidant capacity of cow milk and ewe milk kefir using a direct vat set culture of *Lactococcus lactis*, *Lactococcus cremoris*, *Lactococcus diacetylactis*, *Candida kefir*, and *Saccharomyces unispora*. The average values for DPPH throughout fermentation in E-GR (ewe milk kefir grain) and E-CS (ewe milk culture strains) were 1.68 and 9.05 mg of Trolox equivalents (TEs)/100 mL, respectively, whereas the DPPH values for C-GR (cow milk with kefir grains) and C-CS (cow milk with culture strains) were 3.99 and 9.57 mg of TEs per 100 mL, respectively. The average ABTS scavenging capacities in E-GR and E-CS were 11.34 and 32.93 mg of TE/100 mL, and in C-GR and C-CS were 10.25 and 22.44 mg of TE/100 mL, respectively. Bensmira and Jiang [39] have produced peanut milk kefir from skimmed milk using a commercial freeze-dried kefir starter culture. In the same study, two antioxidant tests (DPPH and FRAP) were performed, and it was found that the scavenging activity of peanut milk and peanut milk kefir increased significantly in parallel to the concentration from 5 to 35 mg/mL.

In addition, according to Kansci et al. [40], radical-scavenging peptides are produced during the fermentation of bovine milk or its digestion in the gastrointestinal tract by *β*-casein (*β*-CN). Camel milk contains 65% of *β*-CN, which makes it rich in antioxidant properties [41, 42]. Soleymanzadeh et al. [43] evaluated the antioxidant properties of camel and bovine milk inoculated with various strains. In this study, the following strains of bacteria were examined: *Weissella cibaria*, *Enterococcus faecalis*, *Leuconostoc lactis*, *L. plantarum*, *Lactobacillus paraplantarum*, *Lactobacillus kefiri*, and *Lactobacillus gasseri.* Nine samples were inoculated with *Lactobacillus paracasei* and Leu. *lactis* and were found to be catalase-negative. Bacteria were isolated and identified using a combination of traditional and molecular techniques. The strains were used to ferment both bovine and camel milk for a 24-h period, after which a comprehensive evaluation was conducted. Out of the used strains, DPPH (57.90 ± 4.59* μ*M) and ABTS (1484.35 ± 128.20* μ*M) values were highest in the camel milk fermented by *Leu. lactis*. Studies have shown that camel milk fermented with *L. acidophilus* or *Lactobacillus rhamnosus* PTCC 1637 exhibits significantly higher antioxidant activity compared to unfermented milk and milk from other dairy animals (NRCC, 2013–2014). Moslehishad et al. [44] attributed this enhanced activity to increased proteolytic action during storage, which promotes the release of antioxidant peptides. Similarly, Solanki and Hati [45] reported that *β*-CN in camel milk contributes to the formation of these peptides, further boosting antioxidant potential during fermentation. Overall, the combination of fermentation and storage-driven proteolysis in camel milk leads to the generation of bioactive compounds with superior antioxidant effect. Related to value-added camel milk products, Shori and Baba [46] reported that fermented camel milk with garlic extract had in vitro antioxidant properties.

An increased antioxidant capacity has been reported in cultured goat milk with symbiotics *Limosilactobacillus fermentum* WXZ 2–1 plus *S. thermophilus* and *L. delbrueckii* ssp. *bulgaricus* [47]. In another study [48], the antioxidant capacity of cow, buffalo, and goat milk along with their respective yoghurts have been examined. They found that raw milk contains antioxidants such as free sulfhydryl groups, phenylalanine, tyrosine, and tryptophan. The yoghurts were categorized into two types: with a pure culture (*B. bifidum*) and with mixed cultures (*S. thermophilus* and *L. delbrueckii* subsp. *bulgaricus*). The impact of storage on the bioactive activities of both the yoghurts and the milk was tested under 4°C for 21 days. The analysis suggested that the antioxidant activity observed in raw milk is retained in the stored yoghurts, regardless of the type of culture used. This probably could be due to the natural antioxidants (tocopherol, carotenoids, conjugated linoleic acid [CLA], casein, and lactoferrin) in whey. In a research study [49], the comparison of sheep milk with cow milk has shown blood serum albumin of 0.55–0.6 and 0.3–0.6 g L^−1^, respectively. This serum albumin participate in lipid biosynthesis by binding amino acids, which leads to antioxidant activity. Further, the same authors emphasize the nutritional and biological significance of sheep milk and dairy products, highlighting features such as favorable fatty acid (FA) profiles, milk fat globule size, high CLA, sphingomyelin, fat-soluble vitamins, bioactive peptides, protease–peptone fraction, nucleoside/nucleotide, polyamine contents, minerals, minor compounds like oligosaccharides, and water-soluble vitamins. This concludes that sheep and cow milk products are excellent food sources with many beneficial components transferred to byproducts. Hence, understanding the antioxidant properties of milk and dairy products provides valuable insights into their potential health benefits and underscores the importance of incorporating these nutritious foods into a balanced diet.

### 2.2. The Role of Fermented Dairy Products in Cancer Prevention

Cancer is a great public health issue. Fermented dairy products are gaining recognition for their potential role in cancer prevention, owing to the anticarcinogenic effects exerted by probiotic strains. Microbiota harbor the human body and make it more cancer susceptible to inflammation and inducing DNA damage. However, this subtopic explores how most of the probiotic strains in fermented dairy products exert a potential anticancer effect by improving host gut microbiota beneficial in microbiota modulation, reducing bacteria translocation, and enhancing gut barrier function, anti-inflammatory, and antipathogenic activity. Fermented dairy products significantly decrease bladder, colorectal, and esophageal cancer [45, 50]. The utilization of *Lactobacillus fermentum* in dairy products has been linked to beneficial properties, specifically in reducing the risk of colorectal cancer (CRC) development [51].

**Figure 4:**
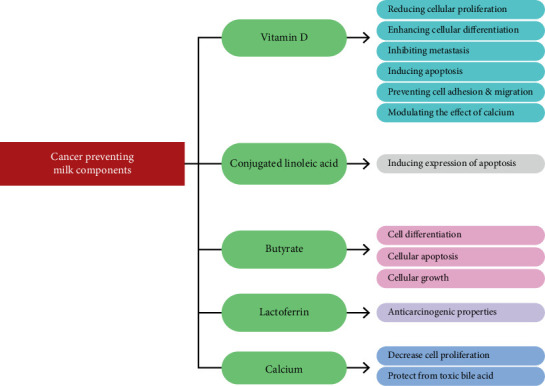
Effect of milk (whole milk and cultured milk) compounds on cancer *Source:* [52, 53].

Dvoodi et al. [52] reviewed the impact of milk and dairy product consumption on cancer risk and concluded that both exogenous and indigenous compounds found in milk (including whole and cultured milk) have the potential to reduce cancer risk (Figure 4).

Colon cancer can be prevented by intestinal bacteria, which also stop procarcinogenic glucuronides from being converted to carcinogens. It has been proven that casein, which makes up about 80% of the protein in cow milk, has anticarcinogenic qualities. Its potential to influence the immune system, particularly by promoting phagocytic activities and raising lymphocytes [53], aids in the prevention of colon cancer. Further, the breakdown of casein during digestion results in the formation of peptides, which exhibit properties that can counteract the mutagenic effect [54]. Other researchers [55] also suggest that those peptides could show anticarcinogenic properties while altering the dynamics of intestinal cell kinetics.

Some prospective studies have shown an inverse relationship between calcium consumption and lowering the incidence of breast cancer [56, 57]. A cohort study conducted by Hjartåker et al. [58] revealed a similar, unfavorable relationship between calcium intake and pre/postmenopausal breast cancer risk. A different study [59] resulted in women who consume 25 g of white cheese daily having a 50% lower risk of developing premenopausal breast cancer than those who consume less than 6 g/day. Concerning bladder cancer, studies have indicated that the consumption of whole milk with high-fat content is positively associated with the risk of developing the disease, in contrast to skim milk and low-fat fermented dairy products.

Due to the metabolites of bacterial starter cultures or their bacterial cells, milk fermentation is also highly helpful in the process of preventing cancer. Additionally, the usage of probiotics considerably increases the beneficial effects of starter cultures. According to research conducted involving Swedish women and men, drinking up to two servings of cultured milk per day reduces the incidence of bladder cancer by 38% compared to those who never drink cultured milk [60]. Probiotic strains *of Enterococcus faecium* RM11 and *L. fermentum* RM28 have inhibited colon cancer cell growth at rates of 21%–29% and 22%–29%, respectively [61]. The findings of a Japanese case-control study on LAB consumption revealed that routine consumption of fermented milk containing the *L. casei* strain lowers the population's risk of bladder cancer [62]. Numerous studies found a negative relationship between drinking cultured milk and the risk of developing various cancers of the colon, bladder, liver, and breast [60, 61, 63, 64]. Yoghurt made from fermented milk is thought to be both healthy and safe [65]. It is thought to contribute to longevity by reducing the formation of harmful substances in the colon. Furthermore, yoghurt consumption may influence the fermentation patterns in the colon, potentially leading to increased production of beneficial short-chain FAs.

Anticarcinogenic properties of yoghurt were reviewed by Fondén et al. [66] mentioning that yoghurt cultures inactivate carcinogens and prevent DNA damage in rats' colon. Moreover, consumption of yoghurt results in suppressing the inflammatory immune response by increasing lymphocytes and immunoglobulin A-secreting cells. Colon cancer, which is highly affected by the role of the diet, has shown good survivability against milk fermented with *L. bulgaricus* than nonfermented milk. Increased risk of colon cancer was linked to elevated activity of many bacterial fecal enzymes involved in the metabolism of genotoxic nitrates [67]. According to Guerin-Danan et al. [68], feeding of infants aged 10–18 months with yoghurt fermented with *S. thermophilus*, *L. bulgaricus*, and *L. casei*, which exhibited reduced fecal glucuronidase activity, could help prevent such fecal enzymatic activities. According to Nagpal et al. [69], *L. acidophilus* reduced the incidence of chemically induced colon cancer in rats fed a diet, and that a possible explanation for these anticancer effects is the inhibition of intestinal bacterial enzymes responsible for converting procarcinogens into more potent carcinogens. Supporting the above, epidemiological data too indicate a link between the consumption of fermented dairy products and a reduced risk of developing some cancers, including colon cancer [67].

Reviewing further, lactobacilli help reduce the overgrowth of harmful bacteria by producing toxic metabolites, which in turn lowers the toxicity of carcinogenic compounds by altering the activity of cancer-related enzymes. Also, urogenital infections and *Helicobacter pylori* infections play a role in competitive exclusion and colonization, which help reduce cancer risk [70]. Moreover, the *Lactobacillus helveticus* R389 strain has been reported to slow the growth of breast tumors [3], while Sharif et al. [71] stated that kefir consumption benefits against several cancer types, such as blood (leukemia), skin, gastric, and colon cancer. This may be due to the presence of high levels of CLA isomers and butyric, palmitic, palmitoleic, and oleic acids in kefir. Further, antiproliferative agents derived from cysteine and cysteine-enriched proteins and peptides or c-glutamyl cysteine dipeptides in cheese have been shown to suppress tumor genesis [72]. Casein, along with its peptides but not free amino acids, has qualities that shield against mutations. In animal studies on colon and breast cancers, whey protein stands out, blocking tumor growth better than other proteins. This strength comes from whey's richness in cystine/cysteine and dipeptides with glutamic acid cystine combos. They rid the body of cell-damaging free radicals and toxins. It keeps proteins stable and ensures the immune system works [73].

It is believed that different types of starter cultures or nonspecific starter bacteria originating from cheese milk could generate biologically active molecules with anticancer properties during cheese ripening. Likewise, the antiproliferative activities are demonstrated by highly ripened cow milk cheeses plus their apoptosis induction in HL-60 cells in contrast to low-ripened cheeses [74]. Moreover, the best oral cancer cell proliferation prevention and improvement in survival rates in oral cancer patients was detected in *Lactiplantibacillus plantarum* out of 21 strains extracted from cheese, milk, and yoghurt [75]. The above results are supported by the study by Rafiq et al. [76] who observed that the water-soluble peptides (WSP) extracted from BCC and CCC exhibited strong growth inhibitory activity against cancer cells. Out of the two WSP extracts, BCC showed relatively higher growth inhibition than CCC. A progressive decrease in the viability of cancer cells, from 30 days to 150 days of cheese ripening, was detected in the study [76]. Generally, these findings highlight the potential of cheese-derived peptides as natural bioactive compounds with anticancer properties, opening possibilities for further research into their mechanisms of action and potential therapeutic applications.

According to Meena et al. [77] donkey milk is rich in antitumor and antiproliferative bioactive peptides. Further, it consists of a whey protein fraction of > 10 kDa that is ideal for cytotoxicity and apoptosis. Not only that, donkey milk also has been tested for the inhibition of breast tumors in mice. The capacity of the separated protein fraction to stimulate the activity of lymphocytes and macrophages makes the possible mechanism of suppression and reduction of the tumor. Lysozyme present in the milk is the major factor for antitumor activity. In addition, sheep milk contains lactoferrin greater than cow milk (0.7–0.9 and 0.02–0.5 g L^−1^) that supports in inhibition of replication, inhibition of angiogenesis, activation of cytotoxicity reactions, and induction of apoptosis, showing a potent anticancer effect [49, 78]. These findings suggest the potential therapeutic value of donkey and sheep milk components in cancer treatment and prevention.

### 2.3. Antimicrobial Properties

**Figure 5:**
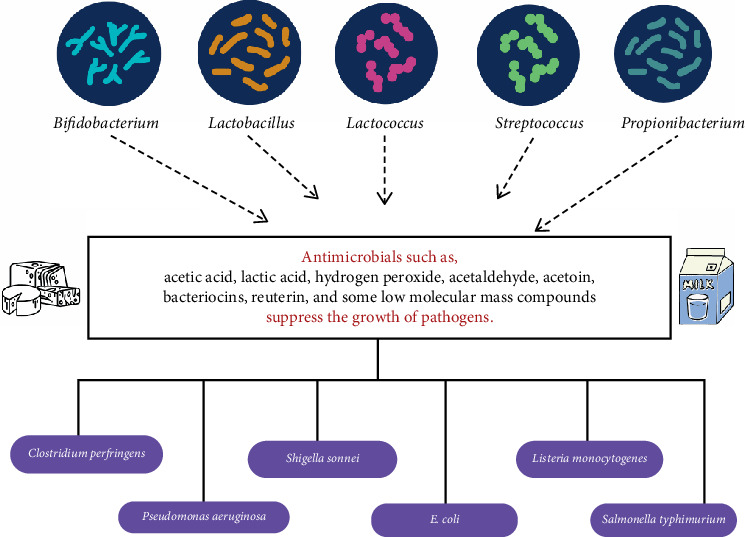
Antimicrobials in milk and dairy products. *Source:* [79, 80].

This subtopic explores the antimicrobial properties inherent in various milk products, particularly those fermented by LAB, highlighting the diverse range of antimicrobial substances produced during fermentation and their effectiveness against a variety of pathogens. Antimicrobial activity is the process of preventing the formulation of microbial colonies or inhibiting the growth of bacteria. As most of the discussed milk products are fermented by LAB, there are two major groups of antimicrobial substances produced, namely, high and low molecular mass substances [79]. It was mentioned that lactic acid, acetic acid, hydrogen peroxide, FAs, acetoin, bacteriocins, and reuterin can be considered a few of the antimicrobials. The antimicrobial effect (Figure 5) of yoghurt describes that lactic acid is the ideal antimicrobial agent as it creates an unfavorable environment for the growth of spoilage bacteria. Bacteriocins, which are proteins or peptides [80] such as curvaticin FS47, carocin lactacin F (against *E. faecalis*), and acidocin (against Gram-positive bacteria) also act as antimicrobials. *L. delbrueckii* ssp. *bulgaricus* and *S. thermophilus* used in dairy products have suppressed the growth of pathogens such as *Shigella*, *Pseudomonas*, *Salmonella*, and *Escherichia coli* using the produced antimicrobials [81].

The composition of the intestinal microbiota can be modulated through the donkey milk administration of probiotic-containing yoghurt, either by introducing beneficial strains into the gastrointestinal tract or by stimulating the growth of existing advantageous microorganisms. Probiotic genera such as *Lactobacillus* and *Bifidobacterium* are known to suppress harmful bacterial species, including *Clostridium perfringens*. Furthermore, *L. plantarum* has been reported to offer protective effects against cellular damage induced by Shiga toxin Type II produced by *E. coli* O157:H7, as well as by extracellular factors secreted by *Bacillus cereus* [82].

As an alternative to cow milk, buffalo milk yoghurt has been prepared with *L. rhamnosus* GG, incorporated with bael (*Aegle marmelos*) fruit pulp to investigate its quality attributes and storage stability [83]. According to the study, the viable count of probiotics in buffalo milk yoghurt remained above 10^7^ CFU/g throughout 21 days of storage, suggesting that it is highly effective in delivering therapeutic effects while maintaining viability. Further, the addition of bael extracts (5% the best incorporation) had a positive effect on the growth of *L. rhamnosus* GG, but 10% of fruit pulp incorporation to buffalo yoghurt has shown a reduction in the viability of probiotics after 14 days, and authors suggest that it may be due to the antimicrobial effect of phenolic compounds. The results of Wijesekara et al. [34], in which they studied natural color-enriched probiotic stirred yoghurt, concluded that phenolics have potential antimicrobial properties. According to their data, the turmeric incorporation in stirred-yoghurt with thermophilic cultures and *B. bifidum* has shown an adverse effect on the growth of the starter culture while anthocyanin from blue pea and *Hibiscus* incorporations separately facilitated the metabolic rate and growth of starter cultures. Moreover, the viability of probiotics over the shelf life was above the accepted standard range (> 106 logs CFU/mL).

Isolation of LAB from Sri Lankan traditional *Meekiri* (milk gel from buffalo [*Bubalus bubalis*] milk) evidenced the presence of *L. fermentum*, *Latilactobacillus curvatus*, *L. acidophilus*, and *Lactiplantibacillus plantarum*. Further, *Meekiri* samples have exhibited strain-specific antimicrobial properties against pathogens like *Listeria monocytogenus* [84]. Prior research [48] investigating the antimicrobial effects of cow, buffalo, and goat milk, as well as their corresponding yoghurts, against *Staphylococcus aureus*, *B. cereus*, *Salmonella typhimurium*, and *E. coli* found that the milk itself exhibited no antimicrobial activity. However, the yoghurts derived from these milks demonstrated notable inhibitory effects against the tested pathogens. Moreover, buffalo yoghurt has shown inhibition only against Gram-positive strains (*S. aureus* and *B. cereus*), while cow milk and goat milk-yoghurt suppressed all tested strains.

When discussing the antimicrobial properties of cheese, previous studies have primarily concentrated on fortified cheese products. In the study by Alexa et al. [85], *Satureja hortensis* L. has been used in both dried and essential oil forms on fresh cows' cheese. According to them, they assessed the antimicrobial properties of the fresh cows' cheese fortified separately with dry plants (in various proportions 0.5–1.5%) and essential oil (0.1%, 0.25%, and 0.5%) on the third and seventh dates, at 30°C. This has resulted in a reduction in the total number of *S. aureus*. The study [86], which was conducted to test the effect of ferulic acid (phenolic phytochemical) in fresh cheese, has shown suppressing characteristics against *Listeria monocytogenes* and *Listeria innocua*. Moreover, the same study stated that nisin, which is used as a preservative in the food industry, depicts inhibitory properties against *L. monocytogenes.* Also, it was reported [87] that *L. fermentum* ME-3, which is used as a probiotic in cheese, can act against enteropathogens such as *E. coli*, *S. typhimurium*, and *Shigella sonnei*.

Yirmibeşoğlu and Öztürk [88] in their research studied kefir made of donkey milk and cow milk and revealed the different impacts on the destruction of the bacterial activity against *L. monocytogenes* (clinical isolate), *S. aureus*, *B. cereus*, *Klebsiella pneumoniae*, and *Staphylococcus epidermidis* except for *Pseudomonas aeruginosa*. Further, the activity of *Proteus mirabilis*, which is a clinical isolate, and *E. coli* was suppressed against donkey milk kefir while showing resistance against cow milk kefir. Variations in milk origin may contribute to the differences observed in the above results. Also, kefir has been reported to exhibit antimicrobial effects against Gram-positive *Staphylococcus* and Gram-positive *Bacillus* species [89]. Moreover, various *Lactobacillus* species isolated from kefir grains in different regions (Russia, Turkey, and Spain) have shown antimicrobial activity against enteropathogenic bacteria and have been observed to influence the adhesion of *S. typhimurium* to Caco-2 cells (an immortalized cell line of human colorectal adenocarcinoma cells). In a different study, strains such as *L. cremoris*, *L. lactis*, *S. thermophilus*, and *Streptococcus durans* from Turkish kefir samples were found to inhibit *S. aureus*, while certain strains of *L. lactis* and *L. cremoris* were effective against *E. coli* and *P. aeruginosa*. More recently, in vitro studies have demonstrated the inhibitory effects of kefir on *S. aureus* [90].

Donkey milk and cultured donkey milk have antimicrobial properties that ensure health and well-being among consumers. Meena et al. [77] reported that lactoferrin hydrolyzes the glycosidic bond of mucous polysaccharides, showing its antimicrobial activity in bacterial cell walls. The concentration of lactoferrin in donkey milk is higher (~4×) than in cow milk (Table 3).

According to Ashokkumar et al. [91], donkey milk contains elements that stimulate the proliferation of bacteriocin-producing LAB *L. paracasei*. The bacteriocin produced is effective against various intestinal pathogens such as *Salmonella typhi*, *P. aeruginosa*, and *E. coli*. In support of this, another study by Murua et al. [92] demonstrated that *L. plantarum* cultivated in donkey milk produced a bacteriocin (LP08AD) capable of inhibiting the growth of spoilage-causing bacteria and pathogens, including *Lactobacillus curvatus*, *E. faecium*, and *L. monocytogenes*. Moreover, recent findings indicate that components within donkey milk act as substrates for the proliferation of bacteriocin-producing strains of the *Enterococcus* genus, resulting in the production of three distinct enterocins (Types A, B, and P) that exhibit bactericidal activity against *L. monocytogenes*. Zhang et al. [93] showed donkey milk exhibited the highest sensitivity toward *Shigella dysenteriae* (CGMCC 1.1869) and *Salmonella choleraesuis* (CGMCC 1.1859) strains. Additionally, under in situ conditions, donkey milk demonstrated bactericidal activity against *S. dysenteriae*, reducing the viable count of the sample to below the detection limit. This research has demonstrated that the consumption of fermented dairy products exerts antimicrobial effects by inhibiting enteropathogens, enhancing gut microbiota balance, and producing bioactive metabolites, contributing to improved gastrointestinal health and increased resistance to infections.

### 2.4. Managing Cardiovascular Health With Fermented Milk Products

This section examines the antihypertensive and cholesterol-lowering effects of fermented dairy products, emphasizing their potential in managing cardiovascular diseases such as hypertension and hypercholesterolemia. The discussion is supported by studies highlighting the role of probiotic bacteria in reducing blood pressure and cholesterol levels, along with the influence of milk fermentation on FA profiles and cholesterol metabolism. Hypertension stands as the prevailing cardiovascular ailment, presenting a widespread epidemic that impacts approximately 10%–20% of the adult population and escalates to 40%–50% among individuals aged 50 or above. This condition poses a significant chronic health challenge, linked to various diseases, including arteriosclerosis, stroke, myocardial infarction, and end-stage renal disease [44]. The antihypertensive properties of numerous fermented dairy products have been substantiated through both animal models and clinical trials [94, 95]. The intake of fermented milk or probiotic bacteria has been linked to a reduced risk of increasing blood pressure, and also, *L. helveticus* in fermented milk reduces elevated blood pressure [96] helping in lowering heart diseases.

Moreover, excessive blood pressure leads to hypertension, which can be chronic and degenerative [97]. Over 1 billion people worldwide suffer from this health issue [98]. Additionally, strokes, cerebrovascular accidents, cardiovascular diseases, and renal failures could be the results of hypertension [99]. Angiotensin-converting enzyme (ACE) plays a primitive role in blood pressure, producing Angiotensin II, which is an influential vasoconstrictor, by the conversion of Angiotensin I. Renin–angiotensin is an important metabolic pathway in controlling blood pressure. It is supported by antihypertensive peptides. These peptides are produced in fermented dairy by the proteolysis of endogenous milk enzymes and enzymes from LAB [100]. A study demonstrated that milk fermented with *Lactobacillus* spp. could be a promising treatment for moderate hypertension. This effect is attributed to the production of ACE-inhibitory peptides and gamma-aminobutyric acid [96]. Further, Rahmawati and Suntornsuk [48] supported the above-mentioned argument, stating that antihypertension properties are shown by peptides in milk. Examining the saturated fatty acid (SFA) content in donkey milk (57 g/100 g) and human milk (45 g/100 g), there was a similarity, but it was lower compared to cow milk (71 g/100 g). In the context of unsaturated FAs, donkey milk (43 g/100 g) and human milk (55 g/100 g) have shown higher values than cow milk (29 g/100 g). These values support the nutraceutical properties of the aforementioned milk types by reducing the risk of hypertension and lowering cholesterol, thrombosis, and heart diseases [80].

Serum cholesterol has become the reason for cardiovascular diseases that lead to death. Mechanistically, the consumption of cultured milk has been shown to cause an elevation in human gut bacterial content. These organisms reside in the large intestine and regard to ferment food-derived indigestible carbohydrates, which cause increased production of short-chain FAs that decline circulatory cholesterol concentrations. This occurs either by inhibiting the synthesis of hepatic cholesterol or by redistributing cholesterol from plasma to the liver [101].

Several research studies have been performed using animal models and humans to find out the effect of fermented dairy products on cholesterol concentration [101]. According to Thompson et al. [102], groups of 10–13 healthy volunteers consumed daily servings of 11 different supplements, including 2% milk, whole milk, skim milk, yoghurt, buttermilk, and sweet acidophilus milk over a 3-week period, where it was found that the concentrations of plasma total cholesterol, LDL cholesterol, and HDL cholesterol remained stable in participants despite the increase in calorie intake. Triacylglycerol concentrations were elevated when receiving yoghurt and acidophilus milk during the final period. In another study [103], 30 healthy men in the age range of 33–64 were used to evaluate the test samples: milk that is fermented by yoghurt starters and *L. acidophilus* (plus 0.5% vegetable oil, 2.5% fructooligosaccharides, and 0.5% milk fat) and the control, traditional yoghurt (milk fermented only by yoghurt strains), containing 1% milk fat. During the evaluation, participants were instructed to consume three 125 mL portions of the test or control product with their habitual meals daily. When compared to the control product, the treatment has shown outcomes with lower concentrations of both LDL and total cholesterol.

According to Hosono et al. [104], bile salt hydrolase, an enzyme produced by LAB, catalyzes the hydrolysis of conjugated bile acids to create free bile acids and the appropriate amino acids. These unconjugated bile acids are less efficient detergents for fat solubilization because they are significantly less soluble at low pH than conjugated bile acids. By promoting the production of new bile acids, deconjugation of bile acids can lower serum cholesterol levels in the body. The bile salt deconjugating activity of lactobacilli and *Bifidobacterium* has been demonstrated by Gilliland and Speck [105], Lundeen and Savage [106], Kobashi et al. [107], and Grill et al. [108]. Before dietary cholesterol is absorbed into the bloodstream, *L. acidophilus* is known to be able to lower serum cholesterol [109, 110] by binding dietary cholesterol with the bacteria in the small intestine [111]. Research suggests that the regular consumption of certain probiotics levels down the serum LDL cholesterol [112–114]. It is speculated that probiotic bacteria can metabolize cholesterol and thus reduce its resorption in the gastrointestinal tract. Both in vitro and in vivo experiments have been performed using *Bifidobacteria*, lactobacilli, and other bacteria in milk to show how the bile acid precipitates and deconjugates after the uptake of cholesterol by absorbing them into the membranes [105, 115, 116].

However, fermented dairy products and all the varieties and strains of milk bacteria do not have a hypocholesterolemia impact. This was demonstrated in clinical trials using *E. faecium* and *S. thermophilus* fermented Danish yoghurt named Gaio, which contains the bacteria culture *Lactobacillus* F19. This clinical investigation found that the Gaio yoghurt considerably decreased the amount of cholesterol in a participant's serum (by 8.4%) after 8 weeks of treatment giving 450 mL/day. Yoghurt made with *L. rhamnosus*, *S. thermophilus*, or *L. acidophilus* was tested concurrently in the same trials, but none of these three bacteria displayed a positive effect of lowering the cholesterol [114]. It has been demonstrated that consuming fermented dairy products regularly for 6 months improves the LDL/HDL ratio and raises serum HDL levels in 29 women aged between 19 and 56 years who had no coronary heart disease or diabetes and were not taking any medication that affected the blood lipid mechanism [117]. According to the analysis, individual isolates of the 166 *Enterococcus* and *Lactobacillus* strains found in unpasteurized Bryndza (classic Slovak Liptauer sheep milk cheese) metabolize cholesterol by 12%–56% [112]. Clinical research on participants who consumed (100 g/day) Bryndza for 8 weeks revealed a significant reduction in both total and LDL cholesterol levels. The people with higher initial cholesterol readings experienced the greatest reduction [112].

Furthermore, a review [45] outlined several proposed mechanisms by which camel milk may help regulate cholesterol levels. Fermented camel milk, specifically containing *Bifidobacterium lactis* (BB-12), has been demonstrated to possess a hypocholesterolemic effect through in vivo experiments in rats and the lowering of plasma and liver cholesterol levels. In general, microbes involved in fermentation should be resistant to bile and capable of deconjugating and binding bile acids and cholesterol in order to help reduce the risk of cardiovascular diseases [118].

Despite buffalo milk being high in fat content, it typically contains lower levels of cholesterol compared to cow milk, which renders buffalo milk comparatively less risky for health concerns associated with cholesterol consumption [84]. In the Indian food context, buffalo milk gels (*Dadhi*) cultured with *L. fermentum* I-11 and *Leu. lactis* subsp. *lactis* I-2775 have shown the capability to bind cholesterol [119].

The FA profile of goat milk probiotic yoghurt (GPY) and cow milk probiotic yoghurt (CPY) was analyzed [120] and resulted in a higher quantity of long-chain FAs in both samples. Myristic acid and stearic acid were found to be higher in GPY. Medium-chain FAs which are depleting the body's cholesterol storage (such as capric acid and caprylic acid) and polyunsaturated FAs were also significantly higher in GPY. Additionally, GPY contained a total of eight monounsaturated fatty acids (MUFA), whereas only four were identified in CPY. MUFA has been stated to be a beneficial FA in reducing cholesterol levels. These findings offer valuable insights into the FA profiles of probiotic yoghurt samples derived from two different milk species. They suggest potential avenues for future research to explore the synergy between probiotic and starter cultures used in yoghurt fermentation and its impact on FA profiles as well as the way it affects lowering cholesterol.

### 2.5. Antiallergen Effects

This subsection explores the antiallergenic effects of different milk processing techniques, the genetic factors that influence allergenicity, and the potential of fermented dairy products such as goat milk, Egyptian cheeses, and Iranian camel milk to reduce allergic reactions. Additionally, it explores the impact of fermented dairy on the microbiome and allergic disease incidence, as well as the immunoregulatory effects of LAB consumption, highlighting their potential in managing allergic conditions such as atopic rhinitis and asthma. Milk allergies occur when the immune system mistakenly identifies certain proteins found in milk as harmful substances, triggering an allergic reaction. The primary proteins in cow's milk responsible for allergic reactions are casein and whey proteins. When a person with a milk allergy consumes milk or milk products containing these proteins, their immune system reacts by producing antibodies, such as immunoglobulin E (IgE), which leads to the release of histamine and other chemicals. This immune response can cause various allergic symptoms, including skin rash, hives, digestive problems, and in severe cases, anaphylaxis .

Fermentation of milk alters the composition of milk and modifies its proteins. During fermentation, certain proteins in milk, including casein and whey proteins, may undergo structural changes. These changes can alter the shape and properties of the proteins, potentially reducing their allergenicity. For instance, fermentation may break down or modify specific protein epitopes (regions recognized by the immune system), making them less likely to trigger an allergic response [121].

**Figure 6:**
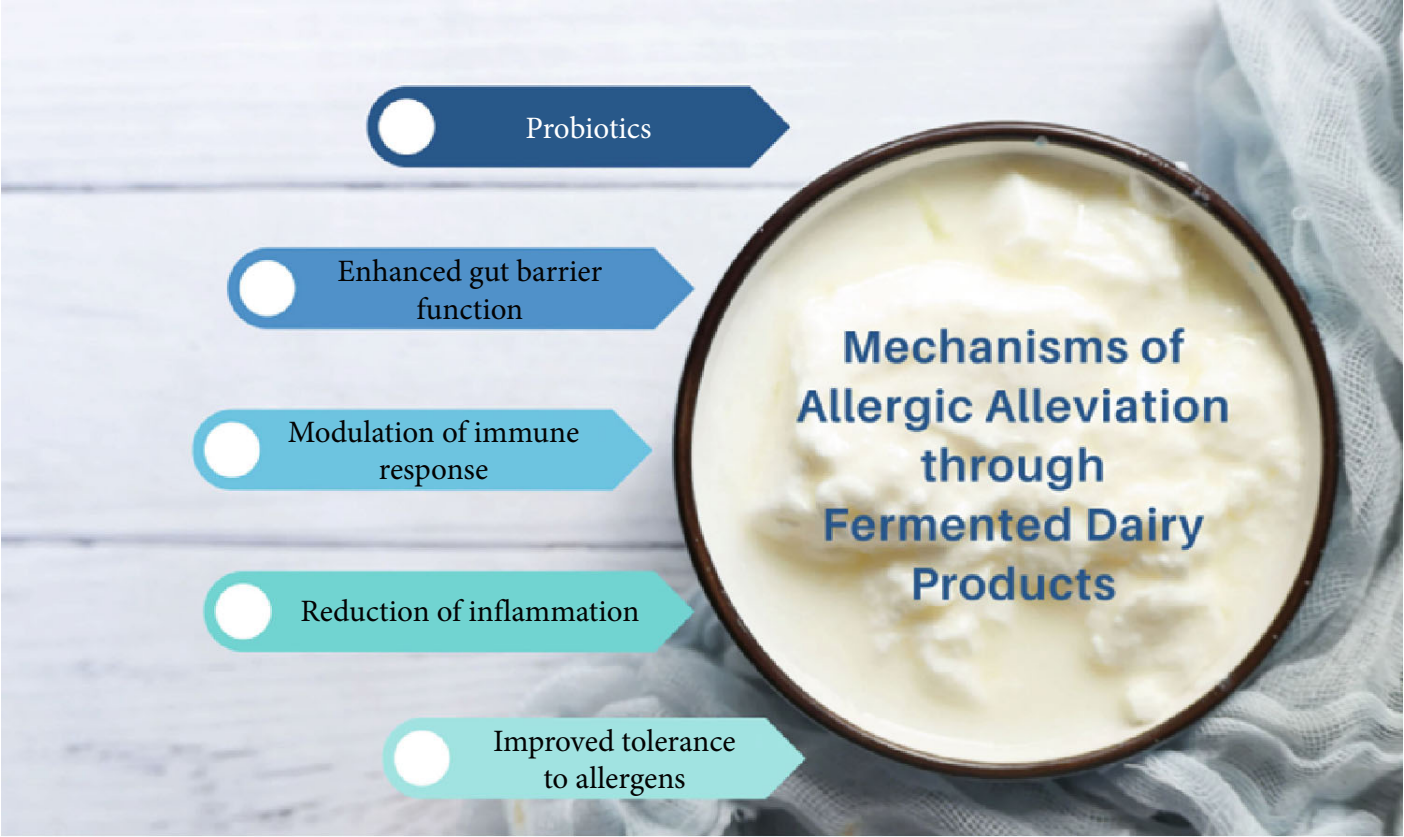
Multiple mechanisms in fermented dairy products for alleviating allergies.

Fermentation involves the activity of enzymes produced by bacteria, which can break down complex proteins into smaller peptides and amino acids. This enzymatic breakdown may reduce the size of protein fragments that could potentially produce an allergic reaction. Smaller protein fragments are less likely to stimulate the immune system and trigger allergic responses. Fermented dairy products contain probiotics and other mechanisms, which can help to modulate immune responses as illustrated in Figure 6.

**Figure 7:**
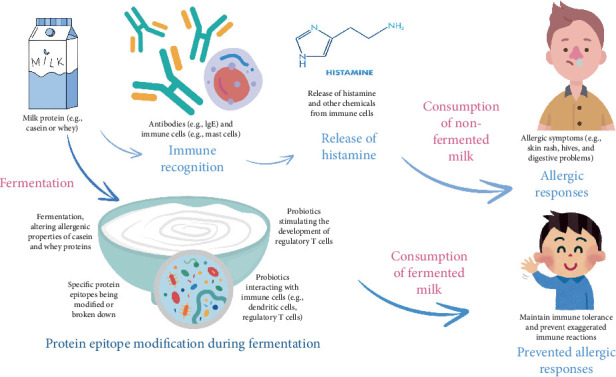
Impact of biotransformations in fermented dairy products on the reduction of milk allergies.

Probiotics may promote the development of regulatory immune cells, such as regulatory T cells, which play a role in maintaining immune tolerance and preventing exaggerated immune reactions, including allergies [122, 123]. By promoting immune tolerance, probiotics in fermented dairy products may mitigate allergic responses (Figure 7) to milk proteins.

Consequently, fermented dairy products may be better tolerated by individuals with milk allergies compared to unfermented milk. However, individuals with severe milk allergies need to exercise caution and consult healthcare professionals before consuming fermented dairy products to ensure safety and suitability.

**Figure 8:**
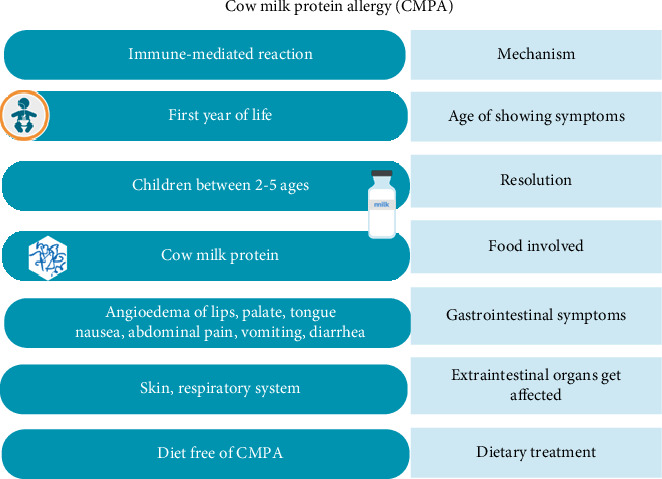
Cow's milk protein allergy in children below 6 years of age.

The prevalence of cow's milk protein allergy (CMPA) varies significantly between studies, and several factors contribute to this variability (Figure 8). Variations in diagnostic criteria, study populations, geographical locations, and methodologies all have an impact on the reported prevalence rates [124]. According to the statement provided by Bavaro et al. [124], the prevalence of CMPA is reported to be 0.6%–3% among children under the age of 6 years (Figure 8) and 0.3% among older children/teenagers, while less than 0.5% among adults.

This could be due to factors such as the maturation of the immune system, changes in dietary habits, and the potential development of tolerance over time. Liang et al. [125] stated that CMA is related to cow milk proteins (CMPs) where *β*-lactoglobulin (*β*-LG) and *α*-lactalbumin (*α*-LA) are the main allergens. The same authors reported that fermentation, high-pressure processing, ultrasound, thermal processing, glycation, enzymatic hydrolysis, and irradiation-like processing methods help in reducing CMA. They further focused on the effect of alcalase hydrolysis and *L. paracasei* fermentation in cow milk on the antiallergen properties. It was concluded that there is a significant reduction of 25.71%–73.37% in antigenicity through fermentation. Compared to cow milk, goat milk possesses lower allergenicity due to the presence of *α*S1-CN genetic polymorphism [126]. This was proved by running individual milk samples from 25 goats possessing varying *α*S1-CN genotypes using SDS-PAGE and immunoblotting techniques with monoclonal antibodies targeting bovine *α*-CN and sera obtained from children with cow milk allergies. The findings by Lisson et al. [127] confirmed that differences in allergenicity are based on genetic variants. Moreover, there are also variants of casein. Further, they mentioned that the feeding of goat or buffalo products to CMA patients is limited by the cross-reactivity of IgE antibodies of goats and buffaloes with cow milk caseins. The antiallergen effect of caseins from traditional fermented milk products, such as Egyptian cheeses and Iranian camel milk, is parallel to the above findings [128]. Also, a recent study by Wróblewska et al. [129] demonstrated that the four main milk allergens—*α*-LA, *β*-LG, *β*-CN, and *κ*-casein—could be eliminated from IgE immunoreactivity toward sweet buttermilk by fermenting it with *L. casei* LcY, a LAB species that is widely used in the agroindustry.

The researchers found that fermented buttermilk contained two enzymes that are present naturally in the cell wall of *L. casei* that were able to bind to human IgE from milk-allergic patients [128]. Studies conducted in vitro show that the primary allergens' effective proteolysis can have a direct impact on the antigenicity of fermented dairy products. Also, the proteolytic activity is determined despite the allergens' hydrolytic susceptibility, which is correlated with their structural characteristics. For instance, compact and globular proteins like *β*-LG and *α*-LA are more resistant to gastrointestinal digestion than caseins [130].

Incidences of food allergy and asthma have been recorded in the developed world over the past years. Complexity and heterogeneity of the human microbiome are important factors in developing an allergic disease [131]. *Bifidobacterium* and Group 1 *Lactobacillus* at excessive levels in the children's intestines show a lower incidence of allergic disease [132]. Fermented milk gels like *Meekiri* have the potential to address the challenges faced by those with lactose intolerance. They offer a solution by presenting lower lactose levels, making them more digestible for individuals with lactose intolerance [84]. According to dietary studies, long-term consumption of yoghurt may help adults with atopic rhinitis or nasal allergies by resulting in fewer clinical symptoms, and it lowers their serum IgE levels [133]. On the other hand, Wheeler et al. [134] found that plain yoghurt did not affect immunological parameters or atopy. However, adult asthmatics who consume yoghurt enriched with *Lactobacillus* showed a trend over time toward decreased eosinophilia and higher interferon-gamma production. Moreover, consumption of fermented milk cultures containing LAB has been shown in controlled experiments to increase systemic production of Type I and Type II interferons. LAB has been demonstrated in animal models to increase interferon expression and, in certain situations, to decrease the synthesis of interleukin-4 and interleukins-5 (interleukins needed for the production of IgE) that is triggered by allergens. Recent findings have demonstrated that LABs are strong inducers of prointerferon monokines, such as interleukin-12 and interleukin-18. It is evident, therefore, that there is a strain dependence on the degree of immunoregulation caused by LAB [133]. These findings highlight the potential of fermented dairy products in managing food allergies and asthma, highlighting the importance of the human microbiome and the strain-specific effects of LAB in modulating immune responses.

### 2.6. Protection Against Gastrointestinal Infections

This section explores the effectiveness of probiotics consumed as fermented dairy products in protecting against gastrointestinal infections, including diarrhea, and investigates the potential of fermented dairy products, as well as alternative milk sources such as donkey milk and *Meekiri* (buffalo milk gel), in promoting gastrointestinal health and modulating gut microbiota composition. Diarrhea, like gastrointestinal infections, results from a change in the gut microflora due to an invasion of a pathogen. Replenishing the flora with normal bacteria or with probiotics minimizes this disruptive effect [135]. Saavedra et al. [136] mentioned that the supplementation of infant formulas with *B. lactis* and *S. thermophilus* is protective against nosocomial diarrhea in infants. Rotavirus diarrhea was said to be prevented by the pediatric beverage that contains *Bifidobacterium animalis*, *L. acidophilus*, and *Lactobacillus reuteri*. Further, treatments with *L. rhamnosus* promote a systemic local immune response to rotavirus, which would be important in protecting immunity against infections [137]. Antibiotic-induced diarrhea that includes *Bifidobacterium longum*, *B. animalis* subsp. *lactis*, *L. rhamnosus* GG, *L. acidophilus* La5, and *Streptococcus faecium* and yeast *Saccharomyces boulardii* is being stated to be prevented by the consumption of probiotics [138].

Further, Linares et al. [139] indicated that probiotics can balance intestinal microbiota, regulate appropriate intestinal function, and be useful in the management or prevention of irritable Crohn's illness or irritable bowel syndrome. Even in virus infections of the gastrointestinal tract, there is a strong possibility that probiotics exert immunomodulatory mechanisms. Moreover, LAB could enhance the mucosal immunity in the host while these bacteria are often used in the prevention of diarrhea in farm animals, especially in newborn piglets [140]. Live microbes in fermented dairy in addition to vitamins, minerals, and FAs have the potential to influence immunological responses as well as the composition and functionality of the intestinal microbiota [141, 142]. The disruption and modification of intestinal microbiota may be linked to several reasons; therefore, the scientific community has turned its attention to finding methods for reversing intestinal microbiota dysbiosis and restoring the host's health. Accordingly, it has been determined that the composition of the gut microbiota is significantly influenced by long-term dietary practices as well as particular food components like fiber or phenolics [143, 144]. Further, Chaves et al. [145] and Meyer et al. [146] have reported that yoghurt consumption modulates both humoral and cellular immunity.

*Meekiri* (buffalo milk gel) has been identified as a beneficial option for alleviating health issues like gastritis and constipation, particularly during crucial life stages such as pregnancy, lactation, and periods of illness [87]. According to a recent study [77], raw donkey milk had a low initial microbial load, which contributes to a higher concentration of lysozyme. This makes it suitable for infant feeding, as it improves gastric conditions by preventing or reducing gastrointestinal infections. Moreover, the authors reported that the consumption of donkey milk supports the improvement of gastrointestinal health, irrespective of age, owing to the presence of specific epidermal growth factors and natural antimicrobials in it.

Dalziel et al. [147] investigated the effects of sheep and cow milk, both in unfermented and fermented forms of donkey milk, administered to rats over a 2-week period. Results showed that stomach emptying was more thorough in rats consumed by sheep yoghurt fermented with *S. thermophilus* and *L. delbrueckii* subsp. *bulgaricus* compared to those consumed by cow yoghurt. Additionally, gastrointestinal transit was accelerated in rats treated with sheep milk compared to those treated with cow milk. The noticeable increase in colonic transit with sheep milk compared to cow milk underscores significant species-specific differences, regardless of whether the milk was fermented or not. A study by Rettedal et al. [148] provides insights into how fermented milk positively influences gut microbiota compared to unfermented milk. These researchers also reported that the differences in the animal origin of the milk (cow vs. sheep) may have been influenced by the gut bacteria and proposed that the animal origin exerts a more significant influence on the composition of gut microbes compared to the impact of fermentation.

## 3. Conclusions

Modern lifestyle changes have led to a decline in the consumption of traditionally fermented dairy products, highlighting the need to reevaluate their role in our diets. These products offer more than just unique taste and aroma; they are rich sources of nutrition and health-promoting agents. By carefully selecting bacterial strains and milk types and following appropriate production protocols, manufacturers can maximize their health benefits. Fermentation not only extends shelf life but also enhances nutritional value, while probiotics act as biomedicines, aiding in the prevention and management of diseases such as gastrointestinal disorders, cancers, neurodegenerative diseases, hypertension, and infections caused by pathogenic bacteria. The antioxidant properties of dairy products, derived from sulfur-containing amino acids, vitamins A and E, and carotenoids, are enhanced by the addition of natural additives and specific microbial strains. Compounds like CLAs, lactoferrin, and vitamin D found in milk may help prevent cancers, including colon, premenopausal breast, bladder, and liver cancers. Antimicrobials in fermented dairy products create an unfavorable environment for pathogenic bacteria, protecting human health. Probiotics also help lower blood pressure and cholesterol levels, impacting the FA profile and cholesterol metabolism. However, allergic reactions to casein and whey proteins in milk can cause immune responses, making it crucial to address potential allergens. Probiotics are especially effective in preventing gastrointestinal diseases like diarrhea by altering gut microflora. Further research is required to fully understand the antihypertensive, antiallergenic, and cholesterol-lowering effects of fermented dairy products. The impact of different milk types on the biofunctional properties of cultured dairy products also needs more exploration. Addressing this research gap will offer valuable insights into maximizing the health benefits of fermented dairy products. The creation of innovative, functional dairy products with validated microorganisms promises long-term health advantages. Integrating fermented dairy products into human nutrition can significantly improve overall well-being. Future studies should aim to develop functional dairy products designed for specific health conditions, ensuring they are accessible and sustainable for populations worldwide.

## Figures and Tables

**Table 1:** Several species of lactic acid bacteria used in the fermentation of milk and milk products.

**Dairy/dairy products**	**Species of lactic acid bacteria used in fermentation**
Cheese, yoghurt, kefir, buttermilk, koumiss, sour cream, and acidophilus milk	*Lactococcus* *Lc. garvieae*, *Lc. plantarum*, *Lc. raffinolactis*, *Lc. piscium*, *Lc. chungangensis*, *Lc. fujiensis*, *Lc. taiwanensis*, *Lc. hircilactis*, *Lc. nasutitermitis*, *Lc. petauri*, and *Lc. formosensis*
	*Streptococcus* *Sc. thermophilus* and *Sc. salivarius*
	*Lactobacillus* *Lb. acidophillus*, *Lb. delbrueckii* subsp. *bulgaricus*, *Lb. delbrueckii* subsp. *lactis*, *Lb. casei*, *Lb. plantarum*, *Lb. rhamnosus*, and *Lb. fermentum*
	*Leuconostoc* *Ln. mesenteroides* subsp. *cremoris*, *Ln. lactis*, and *Ln. citrovorum*
	*Bifidobacterium* *Bifidobacterium bifidum*
	*Enterococcus* *E. faecium*, *E. faecalis*, and *E. durans*

*Note: Source:* [4–17].

Abbreviations: *E*, *Enterococcus*; *Lb*, *Lactobacillus*; *Lc*, *Lactococcus*; *Ln*, *Leuconostoc*; *Sc*, *Streptococcus*; subsp., subspecies.

**Table 2:** Total antioxidant capacity of cow's milk and buffalo's milk cheddar.

**Ripening temperature**	**Ripening days**	**Cow cheddar** **TAC (%)** **M** **e** **a** **n** ± **Sd**	**Buffalo cheddar** **TAC (%)** **M** **e** **a** **n** ± **Sd**
4°C	0	15.42 ± 0.61	20.55 ± 1.46
	40	27.36 ± 1.13	30.72 ± 1.71
	80	40.87 ± 1.59	51.66 ± 2.19
	120	53.42 ± 1.38	73.91 ± 2.34

12°C	0	15.42 ± 0.88	20.55 ± 0.92
	40	32.91 ± 1.33	37.43 ± 1.55
	80	65.41 ± 1.98	77.94 ± 1.28
	120	77.76 ± 1.47	88.30 ± 1.47

*Note: Source:* [35].

Abbreviations: Sd, standard deviation; TAC, total antioxidant capacity.

**Table 3:** Composition of major casein, whey protein fractions, and minor proteins in donkey, cow, and buffalo milk.

**Milk proteins**	**Protein fractions**	**Donkey milk (g kg** ^**−1**^ **)**	**Cow milk (g kg** ^**−1**^ **)**	**Buffalo milk (g kg** ^**−1**^ **)**
Casein	*α*s1-Casein	0.18–0.25	8.00–10.70	0.04–1.68 0.00
	*α*s2-Casein	0.32–0.40	2.80–3.40	
	*β*-Casein	3.90	8.60–9.30	0.04–4.42
	*γ*-Casein	0.00	1.00–2.00	0.00
	*κ*-Casein	0.00	2.30–3.30	0.10–1.72

Whey	*α*-Lactalbumin	1.90	2.00–4.00	1.90–3.40
	*β*-Lactoglobulin	3.30	1–5	0.00

Minor proteins	Protease peptone serum albumin	0.00	6.00–18.00	0.00
		0.40	1.00–4.00	7.16–7.58
		0.37	0.02–0.05	1.50–2.00
		1.00	Trace	0.10–0.90

*Note: Source:* [77].

## Data Availability

All data summarized in the text and tables of this review article were obtained from previously published studies with appropriate attribution cited in the text and tables and complete references provided in the Reference section.
